# Incidence of stroke in the first year after diagnosis of cancer—A protocol for systematic review and meta-analysis

**DOI:** 10.1371/journal.pone.0256825

**Published:** 2021-09-01

**Authors:** Ronda Lun, Danielle Carole Roy, Tim Ramsay, Deborah Siegal, Risa Shorr, Dean Fergusson, Dar Dowlatshahi

**Affiliations:** 1 Division of Neurology, Department of Medicine, Ottawa Stroke Program, University of Ottawa and Ottawa Hospital Research Institute, Ottawa, ON, Canada; 2 Clinical Epidemiology Program, School of Epidemiology, Public Health and Preventative Medicine, University of Ottawa, Ottawa, Ontario, Canada; 3 Division of Hematology, Department of Medicine, Ottawa Thrombosis Program, University of Ottawa and Ottawa Hospital Research Institute, Ottawa, ON, Canada; 4 Department of Education, The Ottawa Hospital, Ottawa, ON, Canada; University of Maryland School of Medicine, UNITED STATES

## Abstract

**Introduction:**

There is an increased risk of stroke in patients with cancer–this risk is particularly heightened around the time of cancer diagnosis, although no studies have systematically quantified this risk in the literature. Patients newly diagnosed with cancer without prior stroke represent a highly susceptible population in whom there is a window of opportunity to study and implement primary prevention strategies. Therefore, the objective of this systematic review and meta-analysis is to identify the cumulative incidence of ischemic and hemorrhagic strokes during the first year after a diagnosis of cancer.

**Methods and analysis:**

MEDLINE, EMBASE, and PubMed will be searched with the assistance from a medical information specialist, from 1980 until present. Eligible studies will include observational studies that have enrolled adult patients newly diagnosed with cancer and report outcomes of stroke during the first year of cancer diagnosis. We will exclude all randomized and non-randomized interventional studies. Data on participant characteristics, study design, baseline characteristics, and outcome characteristics will be extracted. Study quality will be assessed using the Newcastle-Ottawa Scale for cohort studies, and heterogeneity will be assessed using the I^2^ statistic. Pooled cumulative incidence will be calculated for ischemic and hemorrhagic strokes separately using a random-effects model.

**Ethics and dissemination:**

No formal research ethics approval is necessary as primary data collection will not be done. We will disseminate our findings through scientific conference presentations, peer-reviewed publications, and social media/the press. The findings from this review will inform clinicians and patients regarding the risk of stroke in patients newly diagnosed with cancer by quantifying the cumulative incidence of each subtype of stroke during the first year after a diagnosis of cancer. This represents a window of opportunity to implement prevention strategies in a susceptible population.

**Registration ID with Open Science Framework:**

osf.io/ucwy9.

## Background

Cancer remains the leading cause of mortality in Canada, accounting for approximately 30% of all deaths [1]. Comparatively, stroke is the fourth leading cause of mortality in Canada, but remains the leading cause of disability worldwide [2,3]. The combination of cancer and stroke accounts for significant morbidity and mortality, but the relationship between the two diseases is not well understood. While the association between cancer and increased risk for venous thromboembolism has been well established [4], the risk of stroke in patients with cancer is under studied [5].

Multiple registry-based studies have confirmed an increased risk for both ischemic and hemorrhagic stroke in patients with cancer [6–9]. The pathophysiology underlying this increased risk varies for ischemic versus hemorrhagic strokes, but are likely both multifactorial. Postulated mechanisms for ischemic stroke include hypercoagulability from the malignancy, treatment-related adverse effects, and overlapping risk factors (e.g. smoking) [5,8]. Conversely, the most common etiologies responsible for intracerebral hemorrhage (ICH) in patients with cancer are coagulopathy, and hemorrhage of intracranial tumours, which may mimic the presentation and appearance of spontaneous ICH [10].

The incidence of stroke and its temporal correlation with a diagnosis of cancer is variable in the literature. Multiple studies have acknowledged the increased risk for arterial thromboembolic events in the months leading up to a diagnosis of cancer [6,8,9,11]. Andersen et al reported that the risk for stroke tripled around time of diagnosis compared with controls without malignancy [8]. This risk may remain elevated when compared to the general population without cancer, even up to 10 years after a diagnosis of cancer is made [6]. There is also an increased risk for hemorrhagic stroke around the time of cancer diagnosis [8,10]. While a new diagnosis of stroke in those without cardiovascular risk factors should prompt initiation of screening tests for malignancy [12], identifying newly diagnosed cancer patients who have not yet experienced a stroke represents an important population in which primary prevention strategies should be studied. From a clinical perspective, quantifying the risk of stroke *after* a new diagnosis of cancer is important, as it represents a window of opportunity to implement prevention strategies in a susceptible population. A recent systematic review and meta-analysis reported an increased risk for stroke in cancer survivors, but the patient population examined in that study all had “a previous cancer diagnosis” and the temporal relationship between cancer and stroke is unclear [13]. Therefore, to better understand and quantify the relationship between a new diagnosis of cancer and the risk for stroke, the current study aims to examine the risk of stroke immediately following a new diagnosis of cancer–when the risk may be highest. We will conduct a systematic review and meta-analysis of the literature, with the primary objective of identifying the cumulative incidence of ischemic and hemorrhagic strokes during the first year after a diagnosis of cancer.

### Primary objective

To determine the cumulative incidence of stroke (ischemic and hemorrhagic) during the first year after a diagnosis of cancer.

### Secondary objectives

To determine the cumulative incidence of ischemic stroke in newly diagnosed cancer patients.

To determine the cumulative incidence of hemorrhagic stroke in newly diagnosed cancer patients.

To determine the temporal relationship between occurrence of ischemic/hemorrhagic stroke and a new diagnosis of cancer.

## Methods

### Study registration

This study has been registered with the Open Science Framework (osf.io/ucwy9) and will be conducted based on the guidelines of the Cochrane Handbook for Systematic Reviews [14]. This protocol was designed using the Preferred Reporting Items for Systematic Review Protocols (PRISMA-P) guidelines [15]. The final paper will be reported using the updated guideline for Preferred Reporting Items for Systematic Reviews (PRISMA) [16].

### Eligibility criteria

Our comprehensive literature search will address the primary question, “what is the incidence of stroke (ischemic and hemorrhagic) within the first year after a new diagnosis of cancer”? Our search will be limited to adult human subjects (i.e. 18 years or older), since the pediatric population has significantly different risk factors for stroke [17]. We will include all forms of cancer except non-melanoma skin cancer, due to their favourable prognosis and relative inaccuracies in diagnostic coding, which is in line with existing interventional studies in the cancer population [18,19]. Due to the natural history nature of our research question, our search will focus on observational studies only and exclude all interventional studies (including randomized and non-randomized controlled trials), as they represent a different population–it is estimated that less than 5% of adult cancer patients enroll in clinical trials [20], and this population is comparatively much healthier and younger than the general cancer population [21]. A summary of our inclusion/exclusion criteria are provided, and further broken down in terms of subject information vs study type.

### Inclusion criteria

1. Population:
  - Adult human subjects (≥ 18 years)
  - Patients with new diagnosis of cancer, including all cancer types except non-melanoma skin cancers
    - For prospective cohort studies, “new diagnosis of cancer” will be defined as any cancer other than non-melanoma skin cancer that was diagnosed in the 12 months before study inclusion
    - For retrospective or registry-based studies involving cancer patients, we will only extract information on strokes that happened within 1 year after a diagnosis of cancer was recorded
2. Outcomes: need to have well-described and documented strokes as outcomes (including subtype of stroke–ischemic/ICH):
  - For prospective studies:
    - Ischemic stroke definition: neurologic dysfunction caused by focal cerebral infarction confirmed by neuroimaging or pathology [22]
    - Hemorrhagic stroke definition: neurologic dysfunction caused by a collection of blood within the brain parenchyma/ventricles that is not caused by trauma and confirmed by neuroimaging or pathology [22]
  - For retrospective/registry based studies: registry-code based diagnosis for ischemic and hemorrhagic stroke will be used (i.e. International Classification of Diseases)
    - We will record what type of diagnosis codes were utilized by studies. As an example, we have provided the relevant ICD-9 and ICD-10 codes of interest.
    - Ischemic stroke ICD codes:
      ○ ICD-9: 433x, 434x
      ○ ICD-10: I63x
    - Hemorrhagic stroke: ICD codes:
      ○ ICD-9: 431.x
      ○ ICD-10: I61.x
  - However, our search will not be limited to studies using these codes. We will capture case definitions in our extraction form for each individual study.
3. For studies using repeat cohorts/registries, we will assess the relevance of information reported in each publication in addition to the sample size–the study deemed to have the most complete set of variables of interest and largest sample size will be included for analysis
4. Study types:
  - Observational studies only
  - English language

### Exclusion criteria

1. Population:
  - Predominant pediatric population (i.e. >50% of enrolled patients are under the age of 18)
  - Cancer diagnosed > 12 months prior to enrollment in studies:
    - If a study includes a mix of newly diagnosed (≤12 months) cancer patients and patients that were diagnosed after 12 months, only those that were diagnosed ≤12 months prior to enrollment will be used for analysis
    - Retrospective or registry-based studies reporting cancer outcomes above the 1-year cutoff from cancer diagnosis will be included, but only stroke outcomes reported during the 1^st^ year of cancer diagnosis will be included for analysis
2. Outcomes:
  - Subtypes of stroke other than ischemic or hemorrhagic stroke, including cerebral venous sinus thrombosis, aneurysmal or non-aneurysmal subarachnoid hemorrhage, epidural hematoma, subdural hematoma, and transient ischemic attacks
  - Non-descriptive definition of stroke (i.e. “stroke” without specifying the subtype)
3. Study types:
  - Conference abstracts, case reports, case series, editorials, narrative reviews
  - Interventional studies: randomized controlled trials, non-randomized controlled trials, cross-over trials
  - Non-English language

### Information sources

Electronic searches will be conducted in MEDLINE and EMBASE via OVID and PubMed, and will include all relevant studies from 1980 until present. Articles published before 1980 are likely to be irrelevant due to the lack of modern diagnostic imaging technologies used to diagnose stroke and cancer, and is in line with existing literature on similar topics [23]. Our search strategy will be limited to the English language, and studies involving human subjects only. All studies identified for full-text review will undergo further screening of their reference lists for potentially relevant studies.

### Search strategy

Structured search strategies were formulated using MeSH terms for the OVID interface and Emtree terms for the Embase interface after meeting with a medical librarian with expertise in conducting systematic reviews. Full search strategies for all three databases are included in S1 File as examples.

### Study records

#### Data management

Database search results will be imported into Covidence Systematic Review Software (Covidence, Melbourne, VIC, Australia). After removing duplicate results, citation titles and abstracts will be screened by two independent reviewers.

#### Selection process

Two independent reviewers will screen the search results in two stages. The first will be a review of titles and abstracts. Potentially relevant articles will be brought forward for full-text review during the second stage. Discrepancies regarding inclusion of full-text articles will be resolved by a senior third reviewer (DD). A PRISMA flow diagram will be used to summarize the process of study selection.

#### Data collection process

The two reviewers will independently collect data for each phase of the review, including screening, eligibility, and extraction. Once full-text articles are identified for inclusion, each reviewer will also evaluate the completeness, content, and quality of the studies. For any included full-text article that contains missing data, the reviewers will contact the investigators of the original study for clarification. If a same study has multiple reports, the data will be extracted separately but will be collated and linked together for analysis. Data will be extracted from full-text articles using an a priori data extraction form. After extraction has been individually completed, any discrepancies will be resolved via discussion with a senior author (DD) or consultation with a third party, if necessary.

### Data items

Information collected will include:

- Publication data: article title, journal of publication, authorship list, year of publication, country of origin
- Study population: proportion of males/females, average age, baseline vascular risk factors (proportion of patients with hypertension, diabetes, smoking status, dyslipidemia, previous stroke, atrial fibrillation, and heart failure)
- Exposure (cancer): stage, type, location, treatment-related factors (i.e. surgeries, radiotherapy, and use of chemotherapies by drug class)
- Outcomes (stroke): type of stroke, etiology of stroke (i.e. cardioembolic, atheroembolic, cryptogenic, small vessel disease) timing in relation to cancer diagnosis, method of diagnosis (i.e. registry code, imaging, pathology), mortality rates, functional outcomes

### Outcomes and prioritization

The primary outcome we are interested in is stroke–specifically, the subtype of stroke and timing of stroke diagnosis in relation to a diagnosis of cancer. Transient ischemic attacks (TIA) were not included as an outcome of interest, due to low reliability and accuracy in diagnostic coding of TIA, particularly in a non-inpatient setting and when diagnosed by non-experts [24]. Furthermore, the definition of TIA has evolved over the years, which may result in additional heterogeneity [25]. Additional outcomes that may be gathered, if available, include mortality rates and functional outcomes (i.e. modified Rankin Scale).

### Risk of bias in individual studies

Cohort studies will be assessed for methodological rigor at the study level using the Newcastle-Ottawa Scale (NOS) [26], which will be performed by two independent reviewers. Any discrepancies will be settled by consensus after reviewing with a senior author (DD). The NOS includes 3 main domains to assess the quality of observational cohort studies, including selection of study groups, comparability of the groups, and ascertainment of the outcome of interest. The NOS assigns up to a maximum of 9 points for assessment of risk of bias–lower NOS scores indicate greater risk of bias. We plan to perform a subgroup analysis stratified by level of risk of bias, based on the following thresholds for converting the NOS to the Agency for Healthcare Research and Quality (AHRQ) standards:

- Good quality: 3 or 4 stars in selection domain AND 1 or 2 stars in comparability domain AND 2 or 3 stars in outcome/exposure domain
- Fair quality: 2 stars in selection domain AND 1 or 2 stars in comparability domain AND 2 or 3 stars in outcome/exposure domain
- Poor quality: 0 or 1 star in selection domain OR 0 stars in comparability domain OR 0 or 1 stars in outcome/exposure domain

### Data synthesis

Heterogeneity across included studies for primary analysis will be assessed using visual inspection of forest plots and the I^2^ statistic, as recommended by Cochrane Reviews [27]. This represents the percentage of total variation across studies. Heterogeneity is deemed considerable at a level of 50%. Between-study variance will be estimated using a random-effects meta-analysis to produce Tau^2^ values.

We plan on performing sensitivity and subgroup analyses across the following prespecified factors to investigate potential sources of between study heterogeneity: study design (i.e. retrospective vs prospective cohort studies), year of publication, type and stage of cancer (if possible), and studies deemed to have high risk of bias.

### Statistical analysis

#### Outcome analysis

The primary outcomes in this review will be the type and timing of stroke in relation to a new diagnosis of cancer. Type of stroke will be reported as cumulative incidence (proportions) for hemorrhagic vs ischemic strokes. We will report the cumulative incidence of strokes at pre-specified timepoints during the first year after cancer diagnosis (i.e. 1 month, 3 months, 6 months, and 12 months) As this is an incidence study, there will be no comparator vs intervention groups. If mortality data is available, the all-cause mortality rate will be calculated and subgroup analyses will be performed based on the subtype of stroke (i.e. ischemic vs hemorrhagic).

#### Meta-analysis

For each study cohort, we will calculate the cumulative incidence of stroke by using the number of events divided by the total number of people at risk at multiple pre-specified time points during the first year after a diagnosis of cancer (i.e. 1 month, 3 months, 6 months, and 12 months). Due to anticipated heterogeneity in terms of the enrolled populations, we will use a random effects meta-analysis model to pool proportions (cumulative incidence and mortality) from appropriate studies, using generalized linear mixed models [28]. The upper and lower limits of the 95% confidence interval for the proportions at each time interval will be calculated.

All statistical analysis will be performed using Statistical Analysis System (SAS), version 9.4 (SAS Institute, Inc., Cary, North Carolina).

### Meta-biases

For studies included in our primary analysis with an a priori study protocol, we will assess each study individually for potential selective reporting bias. For studies without a published protocol, we will compare the outcomes reported to what is stated in the Methods section. Publication bias will be assessed using funnel plots.

### Confidence in cumulative evidence

We will use Grading of Recommendations Assessment, Development and Evaluation (GRADE) methodology to assess the quality of the evidence for our outcomes [29]. Since our planned systematic review plans on looking at cumulative incidence and does not assess the effectiveness of an intervention, we will adapt GRADE methodology to produce a Summary of Findings (SoF) table, reporting a summary statement for each outcome of interest individually. This will include the number of studies pooled for each outcome, the measure of association with 95% confidence interval, and the certainty of evidence, as summarized using a 4-point scale from very low to high [29]. Patient-important outcomes will be prioritized, therefore, we will report our outcomes in the following order: pooled cumulative incidence of stroke (all subtypes) during the first year, pooled cumulative incidence of hemorrhagic stroke, followed by pooled cumulative incidence of ischemic stroke.

## Ethics and dissemination

The results of this study will help inform clinicians and patients regarding the risk of stroke in patients newly diagnosed with cancer by quantifying the risk of each subtype of stroke during the first year of diagnosis. The findings from this study will be disseminated via conference abstracts/presentations, and the peer-reviewed journal publication process.

## Supporting information

S1 Checklist PRISMA-P (Preferred Reporting Items for Systematic review and Meta-Analysis Protocols) 2015 checklist: Recommended items to address in a systematic review protocol*. (DOC)

S1 File Search strategy. (DOCX)

## References

[1] Statistics Canada. Leading causes of death, total population, by age group. [cited 8 Jan 2021]. Available: 10.25318/1310039401-eng.

[2] Obembe AO, Simpson LA, Sakakibara BM, Eng JJ. Healthcare utilization after stroke in Canada- a population based study. BMC Health Serv Res. 2019;19: 1–8. doi: 10.1186/s12913-018-3827-x 30917828 PMC6438024

[3] Ovbiagele B, Nguyen-Huynh MN. Stroke Epidemiology: Advancing Our Understanding of Disease Mechanism and Therapy. Neurotherapeutics. 2011;8: 319. doi: 10.1007/s13311-011-0053-1 21691873 PMC3250269

[4] Mulder FI, Candeloro M, Kamphuisen PW, Di Nisio M, Bossuyt PM, Guman N, et al. The Khorana score for prediction of venous thromboembolism in cancer patients: a systematic review and meta-analysis. Haematologica. 2019;104: 1277–1287. doi: 10.3324/haematol.2018.209114 30606788 PMC6545838

[5] Navi BB, Iadecola C. Ischemic Stroke in Cancer Patients: A Review of an Underappreciated Pathology. Ann Neurol. 2018;83: 873–883. doi: 10.1002/ana.25227 29633334 PMC6021225

[6] Zöller B, Ji J, Sundquist J, Sundquist K. Risk of haemorrhagic and ischaemic stroke in patients with cancer: a nationwide follow-up study from Sweden. Eur J Cancer Oxf Engl 1990. 2012;48: 1875–1883. doi: 10.1016/j.ejca.2012.01.005 22296948

[7] Cestari DM, Weine DM, Panageas KS, Segal AZ, DeAngelis LM. Stroke in patients with cancer: incidence and etiology. Neurology. 2004;62: 2025–2030. doi: 10.1212/01.wnl.0000129912.56486.2b 15184609

[8] Andersen Klaus Kaae, Olsen Tom Skyhøj. Risk of Ischemic and Hemorrhagic Strokes in Occult and Manifest Cancers. Stroke. 2018;49: 1585–1592. doi: 10.1161/STROKEAHA.118.021373 29866752

[9] Navi BB, Reiner AS, Kamel H, Iadecola C, Okin PM, Tagawa ST, et al. Arterial thromboembolic events preceding the diagnosis of cancer in older persons. Blood. 2019;133: 781–789. doi: 10.1182/blood-2018-06-860874 30578253 PMC6384185

[10] Navi BB, Reichman JS, Berlin D, Reiner AS, Panageas KS, Segal AZ, et al. Intracerebral and subarachnoid hemorrhage in patients with cancer. Neurology. 2010;74: 494–501. doi: 10.1212/WNL.0b013e3181cef837 20142616 PMC2830918

[11] Wei Y-C, Chen K-F, Wu C-L, Lee T-W, Liu C-H, Shyu Y-C, et al. Stroke Rate Increases Around the Time of Cancer Diagnosis. Front Neurol. 2019;10. doi: 10.3389/fneur.2019.00579 31231302 PMC6566310

[12] Rioux B, Keezer MR, Gioia LC. Occult cancer diagnosed following acute ischemic stroke. CMAJ. 2020;192: E1037–E1039. doi: 10.1503/cmaj.200725 32900764 PMC7504883

[13] Zhang F, Wang K, Du P, Yang W, He Y, Li T, et al. Risk of Stroke in Cancer Survivors: A Meta-analysis of Population-Based Cohort Studies. Neurology. 2021;96: e513–e526. doi: 10.1212/WNL.0000000000011264 33277416

[14] Higgins JPT, Thomas J, Chandler J, Cumpston M, Li T, Page MJ, et al. Cochrane Handbook for Systematic Reviews of Interventions. Version 6.1 (updated September 2020). Cochrane; 2020. Available: www.training.cochrane.org/handbook.

[15] Moher D, Shamseer L, Clarke M, Ghersi D, Liberati A, Petticrew M, et al. Preferred reporting items for systematic review and meta-analysis protocols (PRISMA-P) 2015 statement. Syst Rev. 2015;4: 1. doi: 10.1186/2046-4053-4-1 25554246 PMC4320440

[16] Page MJ, McKenzie J, Bossuyt P, Boutron I, Hoffmann T, Mulrow C, et al. The PRISMA 2020 statement: an updated guideline for reporting systematic reviews. MetaArXiv; 2020. doi: 10.31222/osf.io/v7gm2 PMC8008539 33781348

[17] Bigi S, Fischer U, Wehrli E, Mattle HP, Boltshauser E, Bürki S, et al. Acute ischemic stroke in children versus young adults. Ann Neurol. 2011;70: 245–254. doi: 10.1002/ana.22427 21823153

[18] Agnelli G, Becattini C, Meyer G, Muñoz A, Huisman MV, Connors JM, et al. Apixaban for the Treatment of Venous Thromboembolism Associated with Cancer. N Engl J Med. 2020;382: 1599–1607. doi: 10.1056/NEJMoa1915103 32223112

[19] Carrier M, Abou-Nassar K, Mallick R, Tagalakis V, Shivakumar S, Schattner A, et al. Apixaban to Prevent Venous Thromboembolism in Patients with Cancer. N Engl J Med. 2019;380: 711–719. doi: 10.1056/NEJMoa1814468 30511879

[20] Unger JM, Cook E, Tai E, Bleyer A. Role of Clinical Trial Participation in Cancer Research: Barriers, Evidence, and Strategies. Am Soc Clin Oncol Educ Book Am Soc Clin Oncol Meet. 2016;35: 185–198. doi: 10.14694/EDBK_156686 27249699 PMC5495113

[21] Sedrak MS, Freedman RA, Cohen HJ, Muss HB, Jatoi A, Klepin HD, et al. Older adult participation in cancer clinical trials: A systematic review of barriers and interventions. CA Cancer J Clin. 2021;71: 78–92. doi: 10.3322/caac.21638 33002206 PMC7854940

[22] Sacco Ralph L., Kasner Scott E., Broderick Joseph P., Caplan Louis R., Connors J.J. (Buddy), Culebras Antonio, et al. An Updated Definition of Stroke for the 21st Century. Stroke. 2013;44: 2064–2089. doi: 10.1161/STR.0b013e318296aeca 23652265 PMC11078537

[23] Rioux B, Touma L, Nehme A, Gore G, Keezer MR, Gioia LC. Frequency and predictors of occult cancer in ischemic stroke: A systematic review and meta-analysis. Int J Stroke Off J Int Stroke Soc. 2020; 1747493020971104. doi: 10.1177/1747493020971104 33197367

[24] Hall R, Mondor L, Porter J, Fang J, Kapral MK. Accuracy of Administrative Data for the Coding of Acute Stroke and TIAs. Can J Neurol Sci J Can Sci Neurol. 2016;43: 765–773. doi: 10.1017/cjn.2016.278 27426016

[25] Coutts SB. Diagnosis and Management of Transient Ischemic Attack. Contin Lifelong Learn Neurol. 2017;23: 82–92. doi: 10.1212/CON.0000000000000424 28157745 PMC5898963

[26] Wells GA, Shea B, O’Connell D, Peterson J, Welch V, Losos M, et al. The Newcastle-Ottawa Scale (NOS) for assessing the quality if nonrandomized studies in meta-analyses. The Ottawa Hospital Research Institute; Available: http://www.ohri.ca/programs/clinical_epidemiology/oxford.asp.

[27] Higgins JPT, Thompson SG, Deeks JJ, Altman DG. Measuring inconsistency in meta-analyses. BMJ. 2003;327: 557–560. doi: 10.1136/bmj.327.7414.557 12958120 PMC192859

[28] Lin L, Chu H. Meta-analysis of Proportions Using Generalized Linear Mixed Models. Epidemiology. 2020;31: 713–717. doi: 10.1097/EDE.0000000000001232 32657954 PMC7398826

[29] Guyatt GH, Oxman AD, Vist GE, Kunz R, Falck-Ytter Y, Alonso-Coello P, et al. GRADE: an emerging consensus on rating quality of evidence and strength of recommendations. BMJ. 2008;336: 924–926. doi: 10.1136/bmj.39489.470347.AD 18436948 PMC2335261

