# Supplementary material for: Incidence of stroke in the first year after diagnosis of cancer—A protocol for systematic review and meta-analysis
Source: PLoS One. 2021 Sep 1;16(9):e0256825. doi: 10.1371/journal.pone.0256825 (PMC8409607; doi:10.1371/journal.pone.0256825)
Supplement: S1 File — (DOCX) [file pone.0256825.s002.docx]

**SUPPLEMENTAL MATERIALS 1 – SEARCH STRATEGY**

**EMBASE via OVID using EMTree search terms:**

1     malignant neoplasm/ or neoplasm/ or solid malignant neoplasm/ or myeloproliferative neoplasm/ (567513)

2     (cancer or tumo?r* or neoplasms* or carcinoma or leuk?em* or myeloma* or melanoma or hodgkin* or lymphoma or malignanc* or oncology).tw. (5041168)

3     1 or 2 (5128557)

4     exp *brain hemorrhage/ (59384)

5     *cerebrovascular accident/ (84430)

6     brain ischemia/ (150108)

7     ((brain or cerebr*) adj3 (isch?em* or h?emorrhag* or bleed*)).tw. (94262)

8 stroke.ti. (167370)

9 ((isch?em* or h?emorrhag*) adj3 stroke*).tw. (117459)

10 cerebr* vascular accident*.tw. (2269)

11     or/4-10 (379118)

12     3 and 11 (17887)

13     incidence/ (442103)

14     incidence.tw. (1188620)

15     risk*.tw. (3427729)

16     *risk/ or *risk factor/ (150822)

17     13 or 14 or 15 or 16 (4322259)

18     12 and 17 (5883)

**MEDLINE via OVID using MESH search terms:**

1 exp Neoplasms/

2 (cancer or tumo?r* or neoplasms* or carcinoma or leuk?em* or myeloma* or melanoma or Hodgkin* or lymphoma or malignanc* or oncology).tw, kf.

3 1 or 2

4 *stroke/

5 stroke*.tw.

6 ((brain or cerebral) adj isch?em*).tw.

7 ((brain or cerebral) adj h*emorrhage*).tw.

8 Exp Intracranial Hemorrhages/

9 h*emorrhagic stroke*.tw.

10 incidence/ or incidence.tw,kf.

11 risk*.tw, kf

12 risk/ or risk assessment/ or risk factors/

13 10 or 11 or 12

14 4 or 5 or 6 or 7 or 8 or 9

15 3 and 13 and 14

16 case reports.pt.

17 randomized controlled trial.pt.

18 15 not (16 or 17)

19 limit 18 to (English language and humans and yr = “1980 – current”)

**PubMed Search:**

((("Neoplasms"[MeSH Terms] OR ("cancer"[Title/Abstract] OR "tumor*"[Title/Abstract] OR "tumour*"[Title/Abstract] OR "neoplasm*"[Title/Abstract] OR "carcinoma"[Title/Abstract] OR "leukemia"[Title/Abstract] OR "myeloma"[Title/Abstract] OR "melanoma"[Title/Abstract] OR "hodgkins"[Title/Abstract] OR "lymphoma"[Title/Abstract] OR "malignanc*"[Title/Abstract] OR "leukaemia"[Title/Abstract] OR "oncology"[Title/Abstract])) AND ("Stroke"[MeSH Major Topic:noexp] OR "Stroke"[Title/Abstract] OR ("brain ischem*"[Title/Abstract] OR "brain ischaem*"[Title/Abstract] OR "cerebral ischem*"[Title/Abstract] OR "cerebral ischaem*"[Title/Abstract]) OR ("brain hemorrhag*"[Title/Abstract] OR "brain haemorrhag*"[Title/Abstract] OR "cerebral hemorrhag*"[Title/Abstract] OR "cerebral haemorrhag*"[Title/Abstract]) OR "Intracranial Hemorrhages"[MeSH Terms]) AND ("Incidence"[MeSH Terms] OR "Incidence"[Title/Abstract] OR ("Risk"[MeSH Terms] OR "Risk Assessment"[MeSH Terms:noexp] OR "Risk"[MeSH Terms:noexp] OR "Risk Factors"[MeSH Terms:noexp]) OR "risk*"[Title/Abstract])) NOT ("Animals"[MeSH Terms] NOT "Humans"[MeSH Terms])) AND ((english[Filter]) AND (1980:2021[pdat]))
